# Gestational Diabetes Mellitus and Obesity are Related to Persistent Hyperglycemia in the Postpartum Period

**DOI:** 10.1055/s-0040-1721356

**Published:** 2021-01-19

**Authors:** Patricia Moretti Rehder, Anderson Borovac-Pinheiro, Raquel Oliveira Mena Barreto de Araujo, Juliana Alves Pereira Matiuck Diniz, Nathalia Lonardoni Crozatti Ferreira, Ana Claudia Rolim Branco, Aline de Fatima Dias, Belmiro Gonçalves Pereira

**Affiliations:** 1 Obstetrics Departament, Universidade Estadual de Campinas, Campinas, SP, Brazil

**Keywords:** gestational diabetes, obesity, hyperglycemia, postpartum period, overweight, diabetes gestacional, obesidade, hiperglicemia, período pós-parto, sobrepeso

## Abstract

**Objective** To evaluate the obstetric and sociodemographic characteristics of gestational diabetic women who maintained hyperglycemia in the postpartum period (6–12 weeks postpartum).

**Methods** This is a longitudinal cohort study with women who have had gestational diabetes and/or macrosomic children between March 1 ^st^ , 2016 and March 1 ^st^ , 2017. Between 6 and 12 weeks after birth, women who had gestational diabetes collected fasting glycemia, glucose tolerance test, and glycated hemoglobin results. The data were collected from medical records and during an interview in the first postpartum consultation. A statistical analysis was performed using frequency, percentage, Chi-Squared test, Fisher exact test, Mann-Whitney test, and multivariate Poisson regression. The significance level adopted for the statistical tests was 5%.

**Results** One hundred and twenty-two women were included. Most of the women were younger than 35 years old (70.5%), white, multiparous, and with no history of gestational diabetes. Thirteen percent of the participants developed persistent hyperglycemia. A univariate analysis showed that maternal age above 35 years, being overweight, having grade 1 obesity and weight gain under 5 kg was related to the persistence of hyperglycemia in the postpartum period.

**Conclusion** Maternal age above 35 years, obesity and overweight, and the diagnosis of gestational diabetes in the first trimester of pregnancy are associated with hyperglycemia during the postpartum period.

## Resumo

**Objetivo** Avaliar características sociodemográficas e obstétricas de mulheres com diabetes gestacional que mantêm hiperglicemia no período pós-parto (6–12 semanas pós-parto).

**Métodos** Este é um estudo longitudinal de coorte com mulheres com diagnóstico de diabetes gestacional e/ou macrossomia fetal entre 1° de março de 2016 a 1° de março de 2017. As mulheres coletaram glicemia de jejum, teste de tolerância a glicose e hemoglobina glicada entre 6 a 12 semanas pós-parto. Os dados foram coletados de prontuários médicos e durante entrevista na primeira consulta de revisão pós-parto. Uma análise estatística foi realizada através do cálculo de frequências, porcentagens, teste do qui-quadrado, teste exato de Fisher, teste de Mann-Whitney e regressão multivariada de Poisson. A significância estatística adotada foi de 5%.

**Resultados** Cento e vinte e duas mulheres foram incluídas. A maioria delas tinha menos de 35 anos de idade (70,5%), eram brancas, multíparas, e não tinham história de diabetes gestacional. Treze por cento das participantes desenvolveu hiperglicemia persistente. A análise univariada mostrou que os fatores relacionados com a persistência de hiperglicemia no período pós-natal foram: idade materna acima de 35 anos, sobrepeso, obesidade grau 1 e ganho de peso abaixo de 5 quilos. A análise multivariada incluiu o diagnóstico no primeiro trimestre como fator de risco para hiperglicemia persistente.

**Conclusão** Mulheres acima de 35 anos, obesidade, sobrepeso e diagnóstico de diabetes gestacional no primeiro trimestre estão relacionados com hiperglicemia persistente no período pós-parto.

## Introduction

Gestational diabetes (GD) is a condition in which a woman has increased blood glucose levels detected for the first time during pregnancy and does not meet the diagnostic criteria for diabetes mellitus. 1 It affects from 2.4 to 7.2% of pregnancies in Brazil, and increased rates have been observed due to the epidemic of obesity and overweight. 2

It is estimated that approximately 58% of the cases of diabetes mellitus in Brazil are due to obesity. 3 In pregnant women with GD, higher body mass index (BMI) was associated with type 2 diabetes in the postpartum period. 4

Gestational diabetes is related to maternal and fetal complications, such as neonatal hypoglycemia, macrosomia, fetuses being large for gestational age (LGA), and increased perinatal mortality. 5 The worse the maternal glycemic control, the worse the perinatal results will be. 6

Between 30 and 84% of all women with GD have a recurrence of the disease in future pregnancies, and one third of the patients will maintain postpartum hyperglycemia. 7 8 9 In 2014, Weinert et al. 10 found that 24.1% of women with GD had a diagnosis of diabetes mellitus or impaired glucose tolerance within 6 to 12 weeks postpartum. Persistent hyperglycemia (PH) was associated with family history, a diagnostic 2-h 75g oral glucose tolerance test (OGTT) in pregnancy, insulin use during pregnancy, and C-section. 10

The present study aimed to evaluate the profile of GD women who maintained hyperglycemia during the postpartum period (6–12 weeks) and evaluate the impact of obesity, overweight, and weight gain.

## Methods

We performed a prospective cohort study at the Women's Hospital of Universidade Estadual de Campinas, Brazil, from March 2016 to March 2017. Women with GD and/or LGA fetuses were invited to participate after delivery, and, if accepted, they signed an informed consent form. The women included in the study took part in an interview and had their prenatal card data assessed. Subsequently, women collected fasting glucose, OGTT with 75g of dextrose, and glycated hemoglobin results from 6 to 12 weeks postpartum.

The diagnostic criteria for GD, PH, and diabetes mellitus were established according to the International Diabetes and Gestation Study (IADPSG) and adopted by the American Diabetes Association 7 8 9 : GD is considered when women show fasting glycemia values ≥ 92 mg/dL and/or 75g OGTT with 1h glycemia ≥ 180 mg/dL, and/or 2h glycemia ≥ 153 mg/dL; PH is considered when women show fasting glycemia between 100 and 125 mg/dL and/or OGTT values between 140 and 199 mg/dL; diabetes mellitus is diagnosed when fasting glycaemia is > 126 mg/dL or OGTT values are > 200 mg/dL. 8 Newborns were classified as LGA based on the intergrowth curve.

A statistical analysis was performed with mean and percentages. Chi-Squared or Fisher exact tests were used to compare categorical variables, and the Mann-Whitney test was used to compare numerical variables. Multivariate Poisson regression was performed to evaluate the prevalence ratio to develop PH. The significance level adopted for the statistical tests was 5%, that is, *p* < 0.05.

The Institutional Ethics Review Board approved the study (CAEE: 69791616.8.0000.5404). All research was performed following relevant guidelines/regulations. Informed consent was obtained from all participants.

## Results

We included 177 women, of whom only 122 (69%) underwent laboratory tests, even after phone contact and attempts to reschedule collection. From the 122 women included, 96 had GD diagnosis during antenatal care through altered fasting glycemia values or altered OGTT. Twenty-six women had the diagnosis after birthing babies classified as LGA. None of the 26 women had OGTT during antenatal care as a screening.

Sociodemographic and obstetric characteristics are described in Table 1 . Most women were younger than 35 years (70.5%), white, multiparous, and with no history of GD. Table 2 shows diagnostic and treatment details from the studied population. Almost 50% of the patients had the diagnosis before 12 weeks of pregnancy, and 32.44% were obese. Seventeen (17%) women used insulin during pregnancy.

**Table 1:** Baseline characteristics

Variables	N (%)
Age	
≤ 20 y	13 (10.66)
21–34 y	73 (59.84)
35–39 y	30 (24.59)
≥ 40 y	6 (4.92)
Race	
White	80 (66.12)
Non-white	42 (33.88)
Parity	
Primiparous	34 (27.87)
Multiparous	88 (72.13)
Previous GD	
Yes	13 (10.66)
No	72 (59.02)
Previous macrosomia	
Yes	16 (13.11)
No	65 (53.28)
Previous comorbidities	
Yes	32 (26.23)
No	90 (73.77)
Familial background DM*	
DM 2	76 (63.33)
DM 1	3 (2.50)
None	41 (34.17)

Abbreviations: DM, diabetes mellitus; GD, gestational diabetes.

Missing *2.

**Table 2:** Gestational age at diagnosis, BMI, weight gain during pregnancy and treatment at current pregnancy

Diagnosis	N (%)
Gestational age at diagnosis *	
< 12 w	46 (47)
12–24 w	24 (19.7)
≥ 24 w	30(33.3)
Body mass index**	
Normal weight	27 (24.32)
Overweight	48 (43.24)
Obese I	24 (21.62)
Obese II	12 (10.82)
Weight gain during pregnancy	
≤ 5 kg	42 (34.71)
6–12 kg	45 (37.19)
13–20 kg	31 (25.62)
> 20 kg	4 (2.48)
Treatment	
Diet***	
No	37 (30.58)
Irregular	46 (38.02)
Yes (1,800-2,700 Kcal)	38 (31.40)
Exercises****	
Yes	25 (20.66)
No	82 (79.34)
Insulin*	
Yes	17 (17.00)
No	83 (83.00)

Missing * 22 **11***1****15.

During antenatal care, the majority of the participants (68.60%) did not diet for diabetes properly to treat GD: 30.58% did not follow any diet, and 38.02% did not adhere to dietary recommendations. Regarding physical activity, 25 (20.66%) women reported having performed physical activity during pregnancy.

We found 16 women (13.1%) with PH during the postpartum period; 10 had glycated hemoglobin above 6.1, and 11 had altered OGTT (5 women had glycated hemoglobin AND altered OGTT). The factors related to the persistence were: age >35 years, being overweight, obesity grade 1, and weight gain < 5 kg ( Table 3 ).

**Table 3:** Factors related to postpartum hyperglycemia

	Yes (%)	No (%)	*p* -value*
Age			0.003
≤ 20 y	0	13 (12.26)	
21–34	5 (31.25)	68 (64.15)	
35–39	9 (56.25)	21 (19.81)	
≥ 40 y	2 (12.50)	4 (3.77)	
BMI**			0.049
Normal weight	0	27 (27.27)	
Overweight	7 (46.67)	33 (33.33)	
Obese I	6 (40.00)	18 (18.18)	
Obese II	1 (6.67)	11 (11.11)	
Obese III	1 (6.67)	10 (10.10)	
Weight gain during pregnancy ***			0.026
≤ 5 kg	11 (68.75)	31 (29.52)	
6–12 kg	4 (25.00)	41 (39.05)	
13–20 kg	1 (6.25)	30 (28.57)	
> 20 kg	0	3 (2.86)	

Fisher exact test ** missing 8 *** missing 1.

Table 4 shows the influence of initial BMI, gestational age at diagnosis, diet, and exercises on gestational weight gain. The factors that were related to the lowest weight gain were GD diagnosis in the first trimester, correct diet follow-up, and obesity or being overweight at the beginning of the pregnancy. The performance of physical activity did not show statistically significant weight gain.

**Table 4:** Influence of initial BMI, Gestational age at diagnosis, Diet, and Exercises on gestational weight gain

	Gestational Weight Gain N(%)			
	< 5 kg	6–12 kg	> 12 kg	*p* -value
BMI ^a^				0.030*
Normal weight	3 (7.69)	12 (28.57)	12 (37.50)	
Overweight	14 (35.9)	16 (38.1)	10 (31.25)	
Obese I	11 (28.11)	5 (11.90)	7(21.88)	
Obese II	5 (18.82)	4 (9.52)	3 (9.38)	
Obese III	6 (15.38)	5 (11.90)	0	
Gestational age at diagnosis ^b^				0.002**
< 12 w	22 (59.46)	17 (50.00)	6 (25.00)	
12–24 w	5 (13.51)	11 (32.35)	3 (12.5)	
≥ 24 w	10 (27.03)	6 (17.65)	15 (52.50)	
Diet ^c^				0.023**
No	10 (23.81)	11 (24.44)	16 (48.48)	
Irregular	13 (30.95)	19 (42.22)	13 (39.39)	
< 1,800 Kcal	2 (4.76)	1 (2.22)	0	
1,800–2,200 Kcal	10 (23.81)	6 (13.33)	0	
> 2,200 Kcal	2 (4.76)	4 (8.89)	0	
Exercises ^d^				0.752*
Yes	10 (24.39)	8 (17.78)	7 (20.59)	
No	31 (75.61)	37 (82.22)	27 (79.41)	

*Chi-squared test** Fisher Exact Test; Missing ^a^ 9 ^b^ 27 ^c^ 15 ^d^ 2.

The prevalence ratios of developing PH in the postpartum period are shown in Table 5 . Age ≥35 years, overweight or obesity grade 1, weight gain < 5 kg, previous GD, and performance of adequate diet are related to PH.

**Table 5:** Prevalence ratio of developing persistent hyperglycemia

	PR.* (CI 95%)	*p* -value
Age (years)		
< 35	1.00 (-)	
≥ 35	5.26 (1.83–15.13)	0.002
Parity		
Primiparous	1.00 (-)	
Multiparous	2.55 (0.55–11.80)	0.231
BMI (kg/m ^2^ )		
Normal	1.00 (-)	
Overweight	10.24 (1.01–172.22)	0.023
Obese I	14.56 (1.01–245.60)	0.006
Obese II	6.46 (0.28–148.14)	0.134
Weight gain during pregnancy		
≥ 13 kg	1.00 (–)	–
6–12 kg	3.02 (0.34–27.04)	0.323
< 5 kg	8.91 (1.15–68.98)	0.036
Previous GD		
No	1.00 (–)	
Yes	6.52 (2.43–17.51)	< 0.001
Previous macrosomia		
No	1.00 (–)	—
Yes	2.21 (0.71–6.85)	0.17
Gestational age at diagnosis		
< 12 weeks	3.20 (0.69–14.80)	0.086
12 + 1–23 + 6	4.21 (0.82–21.69)	0.137
≥ 24 weeks	1.00 (-)	–
Diet		
No	1.00 (–)	–
Yes	24.36 (1.49–397.05)	< 0.001
Irregular	7.28 (0.40–130.97)	0.068
Insulin		
No	1.00 (–)	–
Yes	2.81 (0.98–8.08)	0.056

Abbreviation: PR, prevalence ratio.

## Discussion

Our study aimed to investigate PH during the postpartum period among women who developed GD. We found that 13.1% of women with GD maintained hyperglycemia between 6 and 12 weeks after delivery. The main factors associated with PH were age > 35 years, overweight, obesity grade 1, and weight gain < 5 kg during pregnancy.

Among the gestational metabolic changes, increased insulin resistance is observed during pregnancy due to an increase of gestational hormones, such as placental lactogen, cortisol, and progesterone. 5 These physiological changes are intended to guarantee glycemic support to the fetus. 5 Women develop hyperglycemia when increased insulin resistance is not adequately compensated for by increased pancreatic beta-cell insulin production. 11

Data from the literature show discrepancies. Gante et al. 12 found an overall rate of 10.9% of PH after a 6-week follow-up, while Durnwald et al. 13 found a higher rate (31.7%) of PH. On the other hand, Sudasinghe et al. 14 found 21.3% of PH after 6 weeks follow-up and an overall rate of 10% of diabetes mellitus.

In our study, we found that women who were overweight or obese at the start of pregnancy had more chance of developing PH during the postpartum period, while greater weight gain during prenatal care had no influence. On the other hand, we observed that patients who presented a lower weight gain (< 5 kg) were those who maintained hyperglycemia during the postpartum period. Women who were overweight/obese and who had GD diagnosed within the first trimester of pregnancy composed this group. This may justify why we found lower weight gain as a risk factor for PH in our study.

Greater weight gain during pregnancy was also not related to PH in a systematic review involving 95,750 women. 15 Nevertheless, Xiang et al. 16 observed that a greater weight gain during pregnancy was associated with a decrease in the functioning of pancreatic beta cells, which led to increased hyperglycemia. 16

We found that age and obesity/being overweight were the main factors related to PH during the postpartum period. Pastore at al. found that women with GD and a BMI > 25 had a higher risk of developing type 2 diabetes. 4 A systematic review with meta-analysis showed that a BMI > 25, a family history of type 2 diabetes, and advanced maternal age are risk factors for developing type 2 diabetes. 15

One of the limitations of the present study was the time that the women were followed up postpartum. We followed up the women for 6 to 12 weeks, but it is believed that over the years, and with other pregnancies, hyperglycemia or even cases of type 2 diabetes may appear. 14 17 It is important to encourage women to perform diagnostic screening over the years. In a meta-analysis, Bellamy et al. 17 showed a cumulative 60% incidence of type 2 diabetes within 10 years following GD and a 7-fold increased risk of developing type 2-diabetes compared with women without GD. 4 17

## Conclusion

Persistent hyperglycemia between 6 and 12 weeks postpartum is associated with a maternal age above 35 years, a BMI in the overweight and obesity grade 1 ranges before gestation, and diagnosis of GD in the first trimester of pregnancy. Excessive weight gain during pregnancy was not associated with PH.
